# Noise Propagation in Two-Step Series MAPK Cascade

**DOI:** 10.1371/journal.pone.0035958

**Published:** 2012-05-01

**Authors:** Venkata Dhananjaneyulu, Vidya Nanda Sagar P, Gopalakrishnan Kumar, Ganesh A. Viswanathan

**Affiliations:** Department of Chemical Engineering, Indian Institute of Technology Bombay, Powai, Mumbai, India; Centrum Wiskunde & Informatica (CWI) & Netherlands Institute for Systems Biology, Netherlands

## Abstract

Series MAPK enzymatic cascades, ubiquitously found in signaling networks, act as signal amplifiers and play a key role in processing information during signal transduction in cells. In activated cascades, cell-to-cell variability or noise is bound to occur and thereby strongly affects the cellular response. Commonly used linearization method (LM) applied to Langevin type stochastic model of the MAPK cascade fails to accurately predict intrinsic noise propagation in the cascade. We prove this by using extensive stochastic simulations for various ranges of biochemical parameters. This failure is due to the fact that the LM ignores the nonlinear effects on the noise. However, LM provides a good estimate of the extrinsic noise propagation. We show that the correct estimate of intrinsic noise propagation in signaling networks that contain at least one enzymatic step can be obtained only through stochastic simulations. Noise propagation in the cascade depends on the underlying biochemical parameters which are often unavailable. Based on a combination of global sensitivity analysis (GSA) and stochastic simulations, we developed a systematic methodology to characterize noise propagation in the cascade. GSA predicts that noise propagation in MAPK cascade is sensitive to the total number of upstream enzyme molecules and the total number of molecules of the two substrates involved in the cascade. We argue that the general systematic approach proposed and demonstrated on MAPK cascade must accompany noise propagation studies in biological networks.

## Introduction

Biological signaling networks in stimulated cells often transfer information via enzymatic cascades such as Mitogen Activated Protein Kinase (MAPK) cascades. These cascades, ubiquitously found in eukaryotic signaling networks [1], [2] act as key signal amplifiers in many regulatory processes [3]–[6] such as cell proliferation, apoptosis [7]. Proteins involved in MAPK cascades are therefore considered potential targets for multiple diseases [8].

Cells constantly encounter inevitable noise or fluctuations arising due to extrinsic – sources external to cell – and intrinsic – sources internal to the cells – factors. These two types of noise may be correlated under certain conditions [9]. This cell-to-cell variability is a feature that has been observed during many cell-fate processes such as cell division, apoptosis [10]. Fluctuation or cell-to-cell variability or noise flows, along with the signal, into the signaling pathway. While flowing, noise can get amplified/attenuated and therefore, may strongly affect cell’s normal functioning [11]–[13]. In order to maintain normal function, cells must either minimize or take advantage of noise. Propagation and amplification of noise can be beneficial [14]–[21] to cells when it incorporates noise into its functions. Noise propagation has also been reported to be deleterious [22]–[25] in many situations. Thatai and van Oudenaarden [26] showed that, under certain conditions, intrinsic noise attenuates with the number of steps in the transcriptional cascade when the degradation step is a first order process. Shibata and Fujimoto [27] showed using linearization of the Langevin equation formulation that the ultrasensitive signal transduction cascades can result in high amplification of input noise to the cascade.

Undesired attenuation or amplification of fluctuations propagating through MAPK cascade can have a significant impact on the fidelity of the signal and therefore, on the cellular outcome. An understanding of the noise propagation through the cascade can provide vital insights into the conditions under which noise may attenuate or amplify. Such insights can provide clues on the functioning of the cell in the presence of noise. Moreover, it can help devise strategies to control noise propagation in a way that will be beneficial to the cell.

Basic assembly of MAPK cascades consists of several cascade motifs or building blocks [28] such as single-step, series and parallel cascades. Recently, attempts have been made to characterize noise propagation in a few enzymatic building blocks such as single-step [29] and parallel [30] enzymatic cascade. However, noise propagation through series MAPK cascade, an important building block in signaling networks [28] has not yet been characterized systematically. In this study, we consider noise propagation via a two-step series MAPK enzymatic cascade. Conventionally, linearization method applied to appropriate stochastic model of the Langevin type is used for estimating noise in protein cascades [27], [29]–[31]. Using the chosen cascade, we prove that the linearization method (LM) fails to predict the intrinsic noise propagation in MAPK enzymatic cascades. Using global sensitivity analysis, we identify the parameters that have a strong effect on the noise propagation through the cascade.

## Results

### Mathematical model formulation

**Figure 1:**
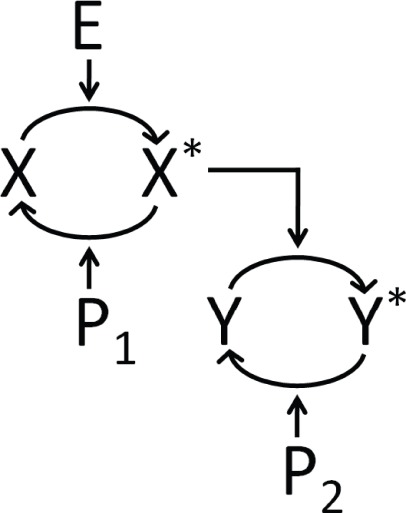
Schematic of a two-step series enzymatic cascade. *X* and *Y* are the unphosphorylated substrates, X^*^ and Y^*^ are the phosphorylated substrates. *E*, *P*_1_, and *P*_2_ are the upstream kinase, and two phosphatases, respectively.

A two step series MAPK enzymatic cascade that appears in Ras/MEK/ERK MAPK cascade [2] is modeled as a sequence of two futile enzymatic steps triggered by an upstream enzyme (Fig. 1). In the first cascade, an upstream enzyme, *E* phosphorylates a substrate *X* to *X* ^*^ and thereby switches it from an inactive state to an active state. Phosphatase *P*_1_, on the other hand dephosphorylates the substrate *X* ^*^ to its inactive state *X*. In the second cascade, *X* ^*^ acts as the enzyme for the phosphorylation of *Y* and *P*_2_ the corresponding phosphatase. The biochemical reactions involved in these four enzymatic actions are
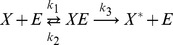
(1)

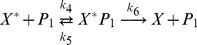
(2)

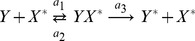
(3)

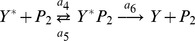
(4)where, 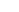
and 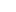 are the phosphorylated substrates. 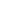,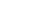, 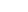 and 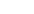 are the reaction intermediates. 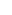 and 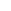, *i = *1 to 6, are the rate constants of biochemical reactions corresponding to the first and second cascades, respectively. The chemical reactions that govern the phosphorylation/dephosphorylation steps in a cell are stochastic in nature [32] and hence we formulate a stochastic model, details of which are presented in Methods section.

We represent the number of molecules of each of the species by the vector 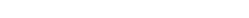. We use smaller case for each of the species to represent the number of that species present in the system. We define the joint probability mass function 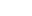, which is the probability that at the instant *t*, 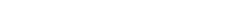, where 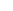 and 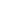 are the total number of molecules of unphosphorylated substrate present in the system at any time *t*, with the initial condition 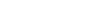. We write the chemical master equation (CME) (Eq. 17 in Methods) to capture the dynamics of 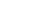. We introduce the Michaelis-Menten type quasi-steady state approximation (QSSA) [33] into the CME (Methods) by assuming the intermediates 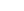,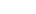, 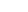 and 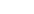 to be fast variables. We then eliminate [31], [33] the fast variables to obtain a reduced CME (rCME) (Eq. 18 in Methods).

### Model predictions

We first consider the linear noise approximation [27], [31] of the rCME obtained using the Ω-expansion [34] (see methods), where Ω is the volume of a cell. Using Ω-expansion of the rCME, we obtain the Langevin type stochastic differential equations (SDEs).
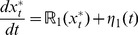
(5)

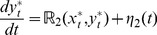
(6)where, 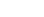 and 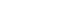 are the rate of formation of the phosphorylated substrates (Eqs (19) and (20) in Methods), and 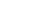
and 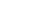 are independent Gaussian white noise terms that have zero mean, that is, 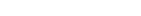 and satisfy Eqs (21) and (22), respectively in Methods. (Note that Eqs (5) and (6) are nonlinear with respect to the number of molecules of the substrates *X* and *Y*.) While rCME permits estimation of only the intrinsic noise, SDEs can be used to estimate both extrinsic and intrinsic noise in the cascade. In the forthcoming sections, we estimate noise using both SDEs and rCME.

### Noise estimation using linearization method (LM)

Linearization of the SDEs, called the linearization method (LM), around a stationary state is a conventional method used to obtain analytical expression for fluctuations [26], [27], [29]–[31] in biological systems. The stationary state 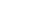 for the macroscopic dynamics was found by solving the macroscopic equations (Eqs 23 and 24) (Methods). The kinetic parameters and initial conditions used in the simulations are presented in Table 1. These consistent set of parameters are based on the quantitative experimental estimates [35] for Ras/MEK/ERK MAPK cascade obtained (using fluorescent probes) for mammalian cells such as HeLa cells and COS7 cells. Notably, the parameter estimates in Fujioka *et al.* [35] has been compared with those from several other reports for different species available in literature. (Parameter values presented in Table 1 are in number of molecules, which, wherever necessary, are converted into concentration by assuming the cell to be a sphere of 10 µM diameter.).

**Table 1:** Biochemical parameters and initial conditions [35].

Initial number of molecules		Phosphorylation reactions		Dephosphorylation reactions	
*x*_0_	757	*k*_3_	0.18 *s* ^-1^	*k*_6_	0.3 *s* ^-1^
*y*_0_	567	*a*_3_	0.22 *s* ^-1^	*a*_6_	0.3 *s* ^-1^
*p*_10_	32	*K*_1_	120	*K*_2_	22
*p*_20_	32	*K*_3_	110	*K*_4_	22
e_0_	94				

**Figure 2:**
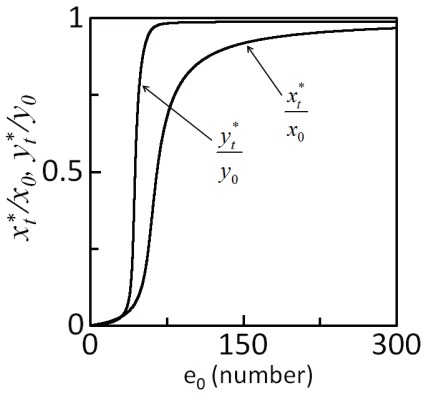
Dependence of the steady state response of the phosphorylated substrates on the total number of the upstream kinase *E*.

Using continuation techniques [36], the steady state response curve that captures the dependence of steady states of the phosphorylated substrates 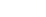
on the total concentration of the upstream enzyme *e*_0_ is obtained (Methods). This response curve presented in Fig. 2 suggests that the phosphorylated substrate quantity is sensitive to the total number of upstream enzyme molecules *e*_0_. The gradual increase in number of molecules of X^*^ and the abrupt increase in that of Y^*^ with change in *e*_0_ is observed due to the signal amplifying nature of the enzymatic cascades [37]. Note that the macroscopic dynamics of a two step enzymatic cascade permits only unique stationary state for any set of parameters, a fact verified by Ciliberto *et al*. [38] using Advanced Deficiency theory [39]. Therefore, the cascade cannot exhibit a bistable behavior.

Next, we linearize the SDEs (Eqs 5 and 6) around the stationary state 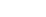 and *e*_0_, and obtain the set of dynamic equations for the perturbations 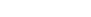, 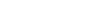. In addition to the perturbations 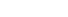, we also introduce perturbation 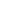 to the mean number of enzyme *E*. The linearized equations (Methods) are
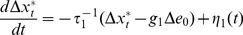
(7)

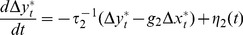
(8)where, 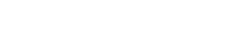 and 
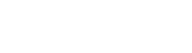
 are the relaxation times, that is the time taken by the system to return to the steady state following a perturbation 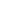 and 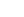. Associated gain factors 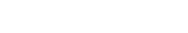 and 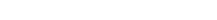 provide an estimate of the response of the phosphorylated substrate to the fluctuations in the total number of enzyme molecules [29], [30]. Note that although the upstream enzyme *E* does not directly participate in the phosphorylation of the substrate *Y*, the fluctuations in the upstream enzyme propagate through the cascade and affect noise in Y^*^.

Fluctuations in X^*^ and Y^*^ are then obtained by simultaneously solving Eqs (7) and (8) using Fourier transforms (Methods and Text S2). Total noise in the system around the steady state [29] is given by the square of the appropriate perturbations, which is a sum of the extrinsic noise 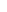 and intrinsic noise 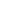. In this study, we assume that the intrinsic and extrinsic noise have independent noise sources. Assuming Poisson statistics for the birth and death of the upstream enzyme *E* (via a phosphorylation-dephosphorylation futile cycle) with a time scale of fluctuation τ, we estimate extrinsic noise in the substrates. If 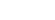
is the fluctuations around *e*_0_, the extrinsic noise in the phosphorylated substrates is given by.
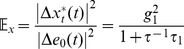
(9)

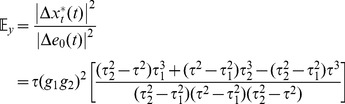
(10)and the corresponding intrinsic noise in the two substrates is given by
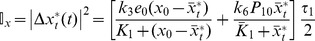
(11)

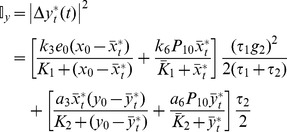
(12)


**Figure 3:**
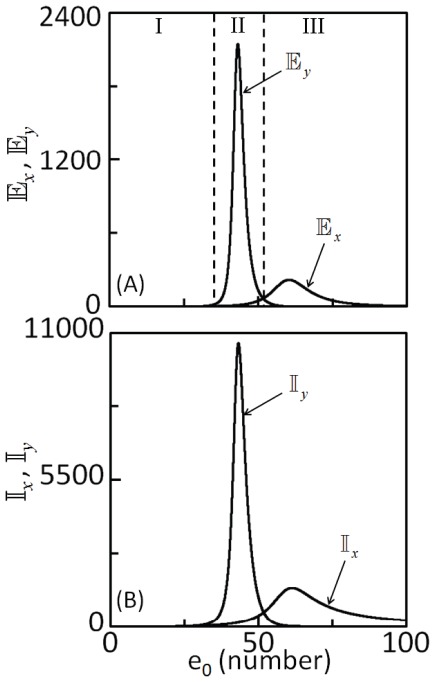
Effect of the total number of molecules of upstream enzyme, *e*_0_ on (A) extrinsic and (B) intrinsic noise in the phosphorylated substrates estimated using the linearization method (LM) applied to the Langevin type stochastic model (Eqs 5 and 6) around the steady state 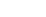
 **.** Parameters used for the simulations are those in Table 1.

The first and second terms in Eq. (12) correspond to intrinsic noise contributions from the first cascade and second cascade, respectively. (The joint probability mass function corresponding to stochastic variables in the linearized model (Eqs 7 and 8) were obtained analytically [40], [41] and is presented in Text S3.) Figures (3A) and (3B) respectively show the effect of the total molecules of enzyme *E* on the extrinsic and intrinsic noise in X^*^ and Y^*^, estimated using Eqs (9) – (12). Based on the relaxation times reported for MAPK cascades [42], [43], we assumed τ = 100s. (Note that τ and the fluctuations in the number of molecules completely describe the strength of extrinsic noise that is input to the cascade.) Figure 3 suggests that the total number of molecules of enzyme controls the amplification or attenuation of total noise, which is the sum of extrinsic and intrinsic noise in the cascade. For the chosen set of parameters, when *e*_0_<∼28 (Fig. 3A, region I) or *e*_0_>∼52 (Fig. 3A, region III), both extrinsic and intrinsic noise propagation in the cascade are almost completely arrested. However, when 28<*e*_0_<52 (Fig. 3A region II), extrinsic noise in Y^*^ is two orders of magnitude greater than that in X^*^, which indicates that noise propagating through the cascade is significantly amplified.

### Intrinsic and extrinsic noise propagation

Noise estimated using the linearization method [27], [29]–[31] shows that the intrinsic noise propagation dominates extrinsic noise propagation (Fig. 3). Linearization method ignores the effects of nonlinearity in the macroscopic rate equations on the estimation of noise propagation. This raises the question as to what extent linearization method, which is well-suited for analytical solution, predicts the extrinsic and intrinsic noise propagation in the cascade. True contributions to the total noise in the substrates from extrinsic and intrinsic noise sources can be estimated only by solving the full nonlinear SDEs, for which analytical solution is non-tractable. Therefore, Euler-Maruyama (EM) method [44] was used to numerically solve the SDEs.

Time-dependent noise terms in the model (Eqs 5 and 6) accounts only for noise contributions due to inherent stochasticity in chemical reactions, that is, intrinsic noise. Therefore, in order to account for extrinsic noise propagation, we introduced perturbations in the total upstream enzyme concentration *e*_0_ by reformulating [45] the SDEs to
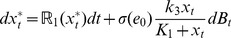
(13)

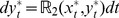
(14)where, σ(*e*_0_), a tunable parameter, represents the strength of the fluctuations in the total upstream enzyme concentration *e*_0_. All effects of the extrinsic noise are incorporated in this tunable parameter. Assuming σ = 0.25, as suggested in literature [45], we conducted extensive stochastic simulations of the reformulated SDEs (Eqs 13 and 14). Concentration trajectory obtained using one simulation mimics the dynamics of the substrates in one cell. Therefore, in order to obtain the trajectory of a population of cells, we conducted 5000 realizations starting from same set of parameters and initial conditions. As one realization corresponds to the dynamics in one cell, 5000 such represent dynamics in those many individual cells in a population. The trajectories of the stochastic simulations were found to be fluctuating around a mean that matches the dynamics obtained using the deterministic formulation (Fig. S1). (Note that this behavior was observed for all values of the total enzyme concentration *e*_0_ considered.) At a certain time where the system attains equilibrium, we estimated the variance in the number of protein molecules in the population, which provides an estimate of the extrinsic noise in the substrates, 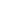 and 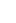. Similarly, we solved Eqs (5) and (6) to obtain intrinsic noise in the substrates, 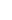 and 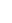. (Note that a vector of independent random numbers generated from a multivariate normal distribution guarantees zero co-variance between the two fluctuation terms in Eqs (5) and (6), respectively.).

**Figure 4:**
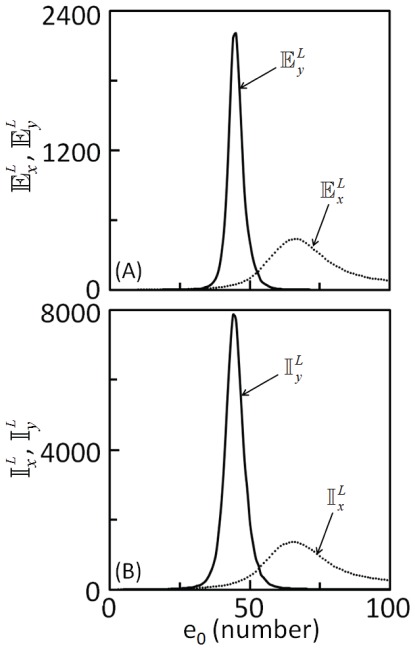
Effect of the total number of molecules of upstream enzyme, *e*_0_ on (A) extrinsic and (B) intrinsic noise estimated by stochastic simulations of the Langevin type stochastic model (Eqs 5 and 6). Extrinsic noise was estimated with σ = 0.25 [45]. 5000 realizations were performed for both extrinsic and intrinsic noise estimates. Parameters used for the simulations are those in Table 1.

Figures (4A) and (4B) show the dependence of extrinsic and intrinsic noise in both substrates, respectively on *e*_0_. Comparison of the Figs (3A) and (4A) suggests that extrinsic noise predicted by solving the SDEs match with those obtained using linearization method. Moreover, the total upstream enzyme concentration ranges at which noise was predicted to attenuate or amplify also agree. This suggests that the linearization method, which provides quick, analytical estimates for noise in enzymatic cascades is a reliable method for extrinsic noise predictions. However, linearization method significantly over predicts intrinsic noise propagation (compare Figs (3B) and (4B)). Actual simulations of the SDEs, though tedious is required to obtain correct estimates of the intrinsic noise. Intrinsic noise estimates which include nonlinear effects (Fig. 4B) preserve the region of noise amplification and attenuation. Moreover, for the chosen set of parameters, comparison of the noise propagation predictions by stochastic simulations of the SDE model (Fig. 4) and the linearization method suggests that the latter method fails to accurately predict intrinsic noise propagation, particularly for the parameters where the steady state response is sensitive to input signal.

**Figure 5:**
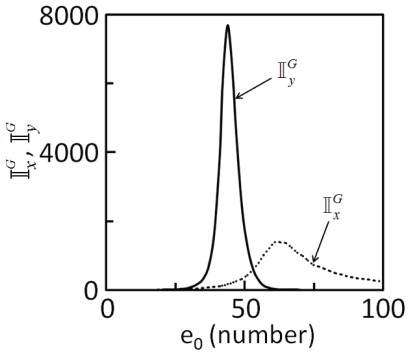
Intrinsic noise in the phosphorylated substrates as a function of the total number of molecules of upstream enzyme, *e*_0_ estimated by Gillespie simulations [**48**]. 5000 realizations were performed. Parameters used for the simulations are those in Table 1.

Noise estimated using stochastic model of Langevin type (Eqs 5 and 6), obtained using linear noise approximation of the rCME [34] (Eq. 18), is valid only up to the order [46] of Ω^-3/2^. In addition to this volume constraint on the region of validity of the estimates, the number of molecules in the cells must be sufficiently large [47]. Therefore, in order to prove that the linearization method fails to make correct predictions, the intrinsic noise estimated by solving SDEs needs to be validated. As the master equation (Eq. 18) is not amenable to analytical approaches, we validate the intrinsic noise predictions using SDEs by performing extensive Gillespie simulations [48], which is a tedious, computationally expensive but an exact method of sampling the trajectories of rCME. Using 5000 realizations of Gillespie simulations, each started from same set of initial conditions and parameters, we estimated the intrinsic noise in the substrates, 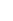 and 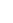. (Note that all the trajectories fluctuated around a mean that matches the the deterministic dynamics (Fig. S1).) Figure (5) shows the dependence of the intrinsic noise in the substrates as a function of the total upstream enzyme concentration. The predictions agree well with those obtained using SDEs (Fig. 4B). A comparison between Figs (3), (4) and (5) also shows that, for enzymatic cascades, the SDEs [34] predicts quite accurately the regions where noise attenuates or amplifies.

When the probability distributions obtained using the three methods were compared, it was evident that the linearization method failed to predict the probability distribution of the two stochastic variables, particularly in the region where the steady state response of the cascade is sensitive. (A comparison of the probability distributions predicted by the three methods for various total upstream enzyme concentration can be found in Text S3 and Fig. S3.) This observation substantiates the finding that linearization method fails to predict the intrinsic noise propagation in enzymatic cascades.

### Sensitivity of intrinsic noise propagation to system parameters

**Figure 6:**
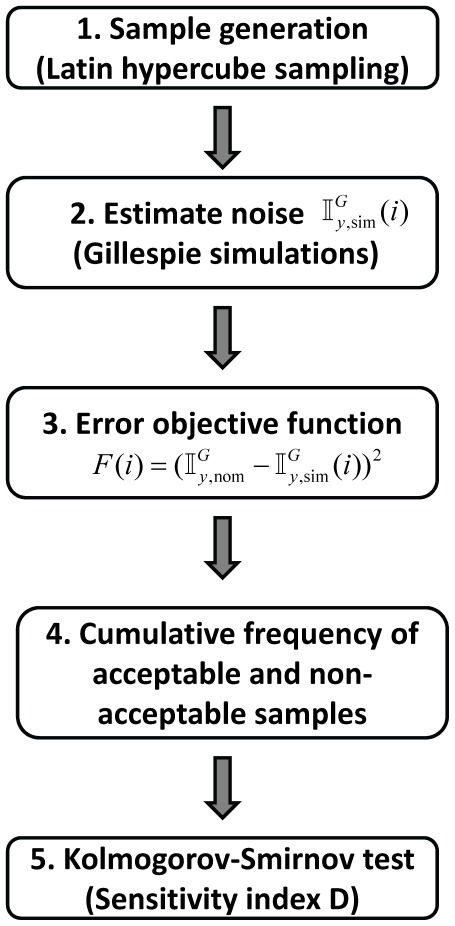
Flow chart describing the steps in the systematic methodology for characterizing noise propagation.

Dynamics of the biochemical reactions involved, and therefore, noise propagation in the cascade is sensitive to the biochemical parameters, that is, rate parameters and initial conditions. Estimates of the biochemical parameters available for MAPK cascade are those measured under *in vitro* conditions and for a certain mammalian species. They are likely to differ not only from one cell type to another but also from one species to another [49]. Therefore, in order to estimate the nature of intrinsic noise propagation at various parameters, we develop a systematic methodology (Fig. 6 and Methods) based on the combination of the global sensitivity analysis (GSA) [50] and Gillespie simulations [48].

Global sensitivity analysis involves estimation of the intrinsic noise, the objective function – for several sets of parameters and use of statistical tools to estimate the relative sensitivity of each of the parameters (Methods). The nature of MAPK cascades and the model considered poses certain constraints on the permitted values of the parameters. QSSA for both substrates is valid [51] only when.
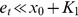
(15a)

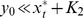
(15b)


Besides, MAPK enzymatic cascades are designed to act as signal amplifiers [3]. Due to this design, the cells are engineered [9], [52] such that, for a two-step cascade considered.
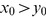
(16)


Note that the total concentration of MEK and ERK in the Ras/MEK/ERK has been experimentally measured for several systems (see Table 1 in Fujioka et al. [35]). The constraint in Eq. (16) is based on the abundance of MEK and ERK in mammalian cells [9], [35], [53]. (Note that this constraint may not be valid for other classes of species [3], [35].).

**Figure 7:**
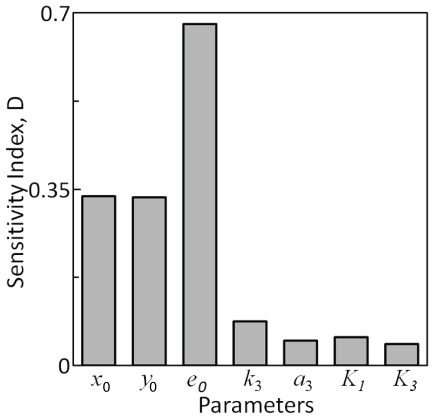
Sensitivity of various parameters towards intrinsic noise in the downstream phophorylated substrate 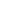. All the parameter sets used in estimating the sensitivity index satisfy Eqs (15) and (16).

We generated 25000 random sets of biochemical parameters using uniform distribution according to the nominal values and corresponding deviations presented in Tables 1 and 2, respectively. From these random sets, we chose those 4820 sets that satisfied the constraints specified in Eqs (15) and (16). Using these 4820 sample sets and the proposed systematic methodology (Fig. 6), we estimated *D*-statistics (Eq. 25), a measure of the sensitivity (Methods), for all the parameters. Figure 7, which presents the *D*-statistics suggests that the intrinsic noise in Y^*^ is sensitive predominantly to *e*_0_, *x*_0_ and *y*_0_. *x*_0_ and *y*_0_ have equal sensitivity towards intrinsic noise propagation. Note that when all 25000 samples were considered for GSA, *e*_0_ still emerged as the key parameter to which intrinsic noise propagation is very sensitive to. However, it is less sensitive to *x*_0_ when compared to that due to *y*_0_ (Fig. S2).

**Table 2:** Parameter range for global sensitivity analysis.

Parameter	Range	Parameter	Range
*x*_0_	4 – 946	*a*_3_	0.144 – 0.216
*y*_0_	4 – 946	*K*_1_	96 – 144
*e*_0_	4 – 473	*K*_2_	96 – 144
*k*_3_	0.144 – 0.216		

## Discussion

Conventionally linearization method [26], [27], [29]–[31] applied to Langevin type stochastic models of signaling cascades such as MAPK cascades is used to estimate extrinsic and intrinsic noise propagation. However, this method fails to predict the intrinsic noise propagation in MAPK enzymatic cascades when the Michaelis-Menten type reaction rates are nonlinear with respect to the substrate concentrations. This failure is predominant in the region where the steady-state response of the cascade is sensitive to the total number of upstream enzyme *e*_0_. We prove this by conducting extensive stochastic simulations of both Langevin type model and the chemical master equation for various ranges of systems parameters. This failure is due to the fact that the linearization method ignores the nonlinear interactions between the enzymatic reaction rates and the fluctuations, which may contribute significantly to the extent of noise propagation through the cascade. On the other hand, the extrinsic noise propagation predicted by the linearization method agrees very well with those obtained by stochastic simulations (Figs 3A and 4A). This agreement can be attributed to two aspects: a) the linear dependence of the rate processes on the total upstream enzyme concentration, b) availability of the tunable parameter σ (Eq. 13) in the stochastic model [45], which captures the fluctuation strength in the upstream enzyme, that is, strength of the extrinsic noise input to the cascade.

Reliable intrinsic noise estimates of the proteins involved in MAPK cascade can only be obtained using complete stochastic simulations of the model equations. Analytical solution of chemical master equation is intractable and the Gillespie simulations are computationally prohibitively expensive. Therefore, not-so-tedious stochastic simulations of the Langevin type linear noise approximation model should be used for a reliable estimate of noise propagation when the number of substrate molecules is sufficiently large. In cases where the number is not sufficiently large, alternative methods proposed by Lan and Popaian [47] may be used to estimate noise in the cascade. In this study, we assume that the extrinsic and intrinsic noise are independent. However, Tănase-Nicola et al. [9] suggested that under certain conditions, the extrinsic and intrinsic noise propagation in an enzymatic cascade may be correlated. It remains an open question as to which method provides a reliable estimate of noise propagation when the two types of noise are correlated.

Studies of noise propagation in activated signaling pathways are limited by the availability of precise information about the kinetic parameters and initial conditions [28]. Identification of the key parameters that strongly affect noise propagation in the pathway can prove useful in designing strategies to control flow of fluctuations in the network. Based on the combination of global sensitivity analysis (GSA) and stochastic simulations, we have developed a systematic methodology (Fig. 6) to identify system parameters to which noise propagation is sensitive. We demonstrated the applicability of the method by identifying the key parameters in the two-step MAPK enzymatic cascade that affect noise propagation. We argue that the proposed systematic methodology, though tedious must accompany noise propagation studies in signaling networks.

Upon applying the proposed systematic methodology, the total number of upstream enzyme molecules *e*_0_ and the total number of downstream substrate molecules *y*_0_ emerged as the two key parameters that affect noise propagation in the cascade. Proteins in MAPK cascades being potential targets [8] for several diseases, identification of the key parameters that affect noise propagation can provide clues for identifying strategies to engineer a cell to commit to a certain desired outcome. In fact, several experimental methods exist to independently alter the total enzyme concentration and substrate concentration in a cell. For example, perturbations in the MAPK cascade can be introduced using siRNA knockdown technique [42]. Besides, specific chemical inhibitors for proteins involved in MAPK cascade [54], [55] can be used to modulate the substrate concentration. An alternative scaffold mechanism [52] can be used to re-wire MAPK cascade [56].

## Methods

### Stochastic model formulation

We represent the number of molecules of each of the species involved in the MAPK cascade (Fig. 1) by the vector 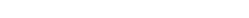, where superscript *t* indicates transpose. We use smaller case for each of the species to represent the number of that species present in the system. We define 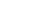 as the joint probability that at the instant *t*, 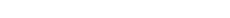, where 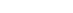 and 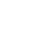 are the total number of molecules of unphosphorylated substrates present in the system at any time *t*, with the initial condition 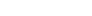. We assume that 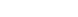, 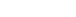, are the initial total number of molecules of *X* and *Y*, respectively. We also assume the conservation relations 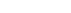, 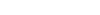, 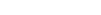, 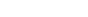, and 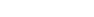 for the total number of molecules of *E*, *P*_1_, *P*_2_, X^*^, and Y^*^, respectively present in the system. Assuming the system to be well-mixed and applying the standard laws of probability, the chemical master equation (CME) [57] that captures the dynamics of the joint probability mass function, 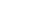 for the set of biochemical reactions involved in the cascade (Fig. 1) is given by.
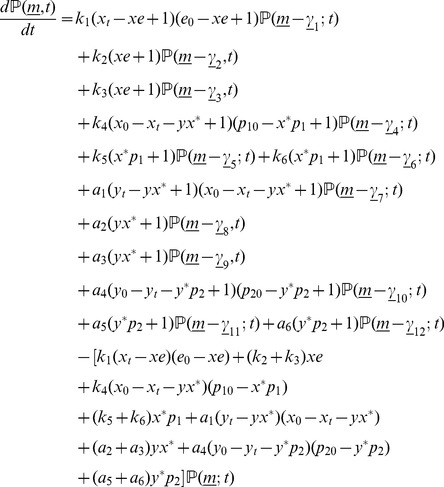
(17)where, 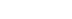 are the stoichiometric coefficient vectors corresponding to the twelve biochemical reactions in Eqs (1) – (4).

### Quasi-steady state approximation

Similar to the implementation of the quasi-steady state approximation (QSSA) in the deterministic framework, we introduce QSSA into the stochastic model (Eq. 17) by assuming the intermediates 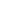,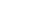, 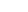 and 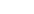 to be fast variables and eliminate them [33]. (Details of the reduction procedure are in Text S1.) The resulting reduced chemical master equation (rCME) is
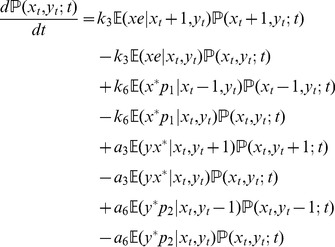
(18a)where,
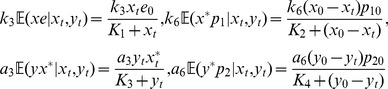
(18b)are the corresponding propensity functions in which the Michaelis-Menten constants of each of the phosphorylation/dephosphorylation reactions are given by 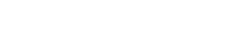.

### Stochastic differential equations (SDEs) model

By applying Ω-expansion [34] to the multivariate rCME (Eq. 18), we derive the SDE model. The SDEs of the Langevin type [58] for the cascade are.
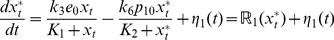
(19)

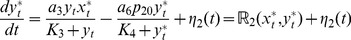
(20)where, 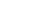 and 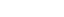 are the net rate of formation of the phosphorylated substrates, and 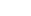
and 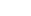 are independent Gaussian white noise terms that have zero mean, that is, 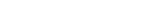 and that satisfy
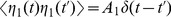
(21)

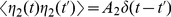
(22)where, 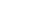 is the Dirac delta function. 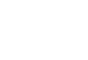 and 
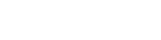
 are the strength of the fluctuations or the total variance of the increment of the respective species in the time interval 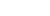 [29]. The strength of the fluctuations is estimated at the mean number of species 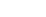 which is the steady state of the macroscopic rate equations. Macroscopic equations obtained by ignoring fluctuations in Eqs (19) and (20), that is, by setting 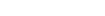 are:
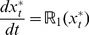
(23)

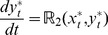
(24)Note that the macroscopic rate equations are also the leading terms [34] in the Ω-expansion of the rCME.

### Response curve

The macroscopic equations (Eqs 23 and 24) were first solved for a set of parameters to obtain a steady state using a Newton solver. Using the solution for this set of parameters as a starting point, the response curve – locus of steady states – was constructed using pseudo-arc length continuation [36]. Programs were written in Matlab® (http://www.mathworks.com).

### Linearization method

Perturbations to the steady state number of phosphorylated substrates 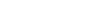, 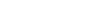 and the perturbations 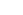 in the mean total number of upstream enzyme *E* were introduced into the model equations (Eqs 5 and 6). The model was then expanded in Taylor series around the base state 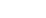 and truncated upto linear terms to obtain the dynamics of the perturbations. This set of linearized equations was solved using Fourier transforms to obtain analytical expressions for the intrinsic and extrinsic noise and thereby, the total noise. (Detailed solution presented in Text S2.).

### Global sensitivity analysis

A flow chart containing the key steps in GSA is presented in Fig. 6. We provide here a brief description of each of these steps:.

1. **Sample generation.** Using Latin Hyper Space sampling technique [59] we generated *S_N_* = 25000 sets of parameters by assuming uniform distribution to each parameter in the set considered.
2. **Noise estimation.** For each set of parameters, we conducted sufficient realizations of Gillespie simulations [48] and estimated noise in the downstream phosphorylated substrate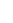.
3. **Error objective function.** For every sample set of parameters, using the 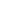, we calculated the error objective function 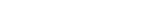, where 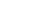 and 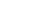 are respectively the noise estimated at the nominal value (Table 2) and those for each *i* = 1 to *S_N_* set of parameters.
4. **Cumulative frequency functions.** A sample is considered acceptable (unacceptable) when 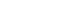 (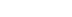) where 
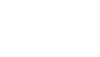
. We then construct the cumulative frequency functions for the acceptable 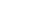 and unacceptable 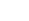 samples.
5. Kolmogorov-Smirnov test: According to the Kolmogorov-Smirnov test [50], the sensitivity of noise in 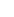, 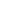 with respect to each of the parameters 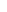 is given by the metric *D* (called D-statistics) defined as
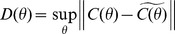
(25)where 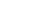 and 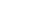 are the cumulative frequency functions of acceptable and unacceptable samples. Higher (lower) the 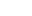 more (less) sensitive is noise in 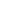 with respect to 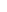.

## Supporting Information

Figure S1
**Dynamics of (A)** 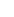 **and (B)** 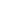 **for the set of parameters in** **Table 1** **.**
(TIF)

Figure S2
**Sensitivity of various parameters towards intrinsic noise in the downstream phophorylated substrate** 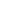 **when all 25000 sample sets of parameters were considered.**
(TIF)

Figure S3
**Comparison of the cumulative probability distribution** 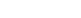 **of the stochastic variables** 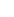 **and** 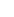 **obtained using the three methods** ***viz.*** **, linearization method (LM), stochastic simulations of SDEs (SDE), and Gillespie simulations (GS) for (A)** ***e*** **_0_  =  44, (B)** ***e*** **_0_ = 70, (C)** ***e*** **_0_ = 150 and (D)** ***e*** **_0_ = 300.**
(TIF)

Text S1 **Quasi-steady state approximation (QSSA) of the chemical master equation.**
(DOC)

Text S2 **Fourier transform method to estimate noise from linearized SDEs.**
(DOC)

Text S3 **Comparison of the probability distributions for all three methods.**
(DOC)
