# Supplementary figures and images for: Noise Propagation in Two-Step Series MAPK Cascade
Source: PLoS One. 2012 May 1;7(5):e35958. doi: 10.1371/journal.pone.0035958 (PMC3341401; doi:10.1371/journal.pone.0035958)

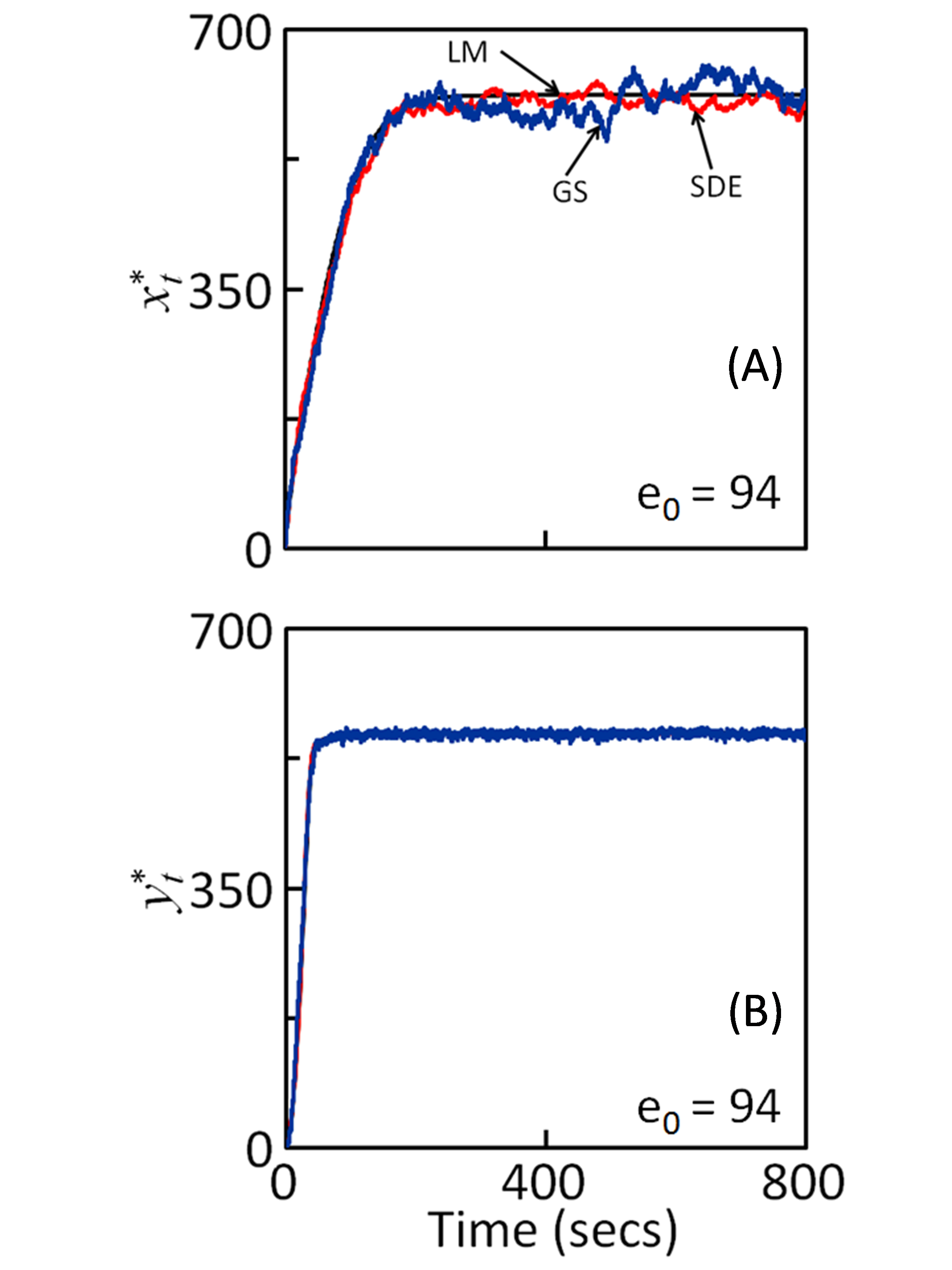

Supplement: Figure S1 — Dynamics of (A) and (B) for the set of parameters in Table 1 . (TIF) [file pone.0035958.s001.tif]

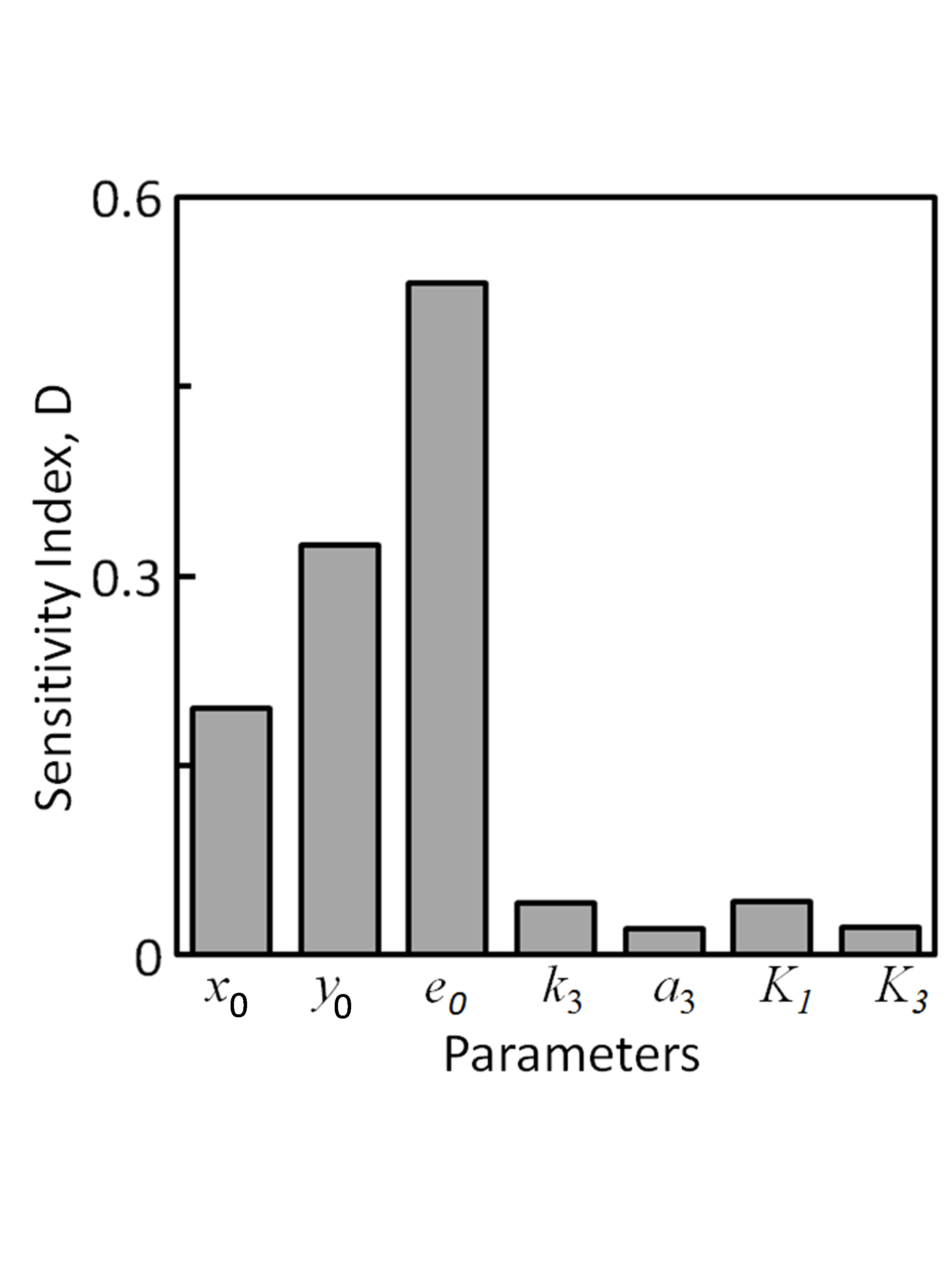

Supplement: Figure S2 — Sensitivity of various parameters towards intrinsic noise in the downstream phophorylated substrate when all 25000 sample sets of parameters were considered. (TIF) [file pone.0035958.s002.tif]

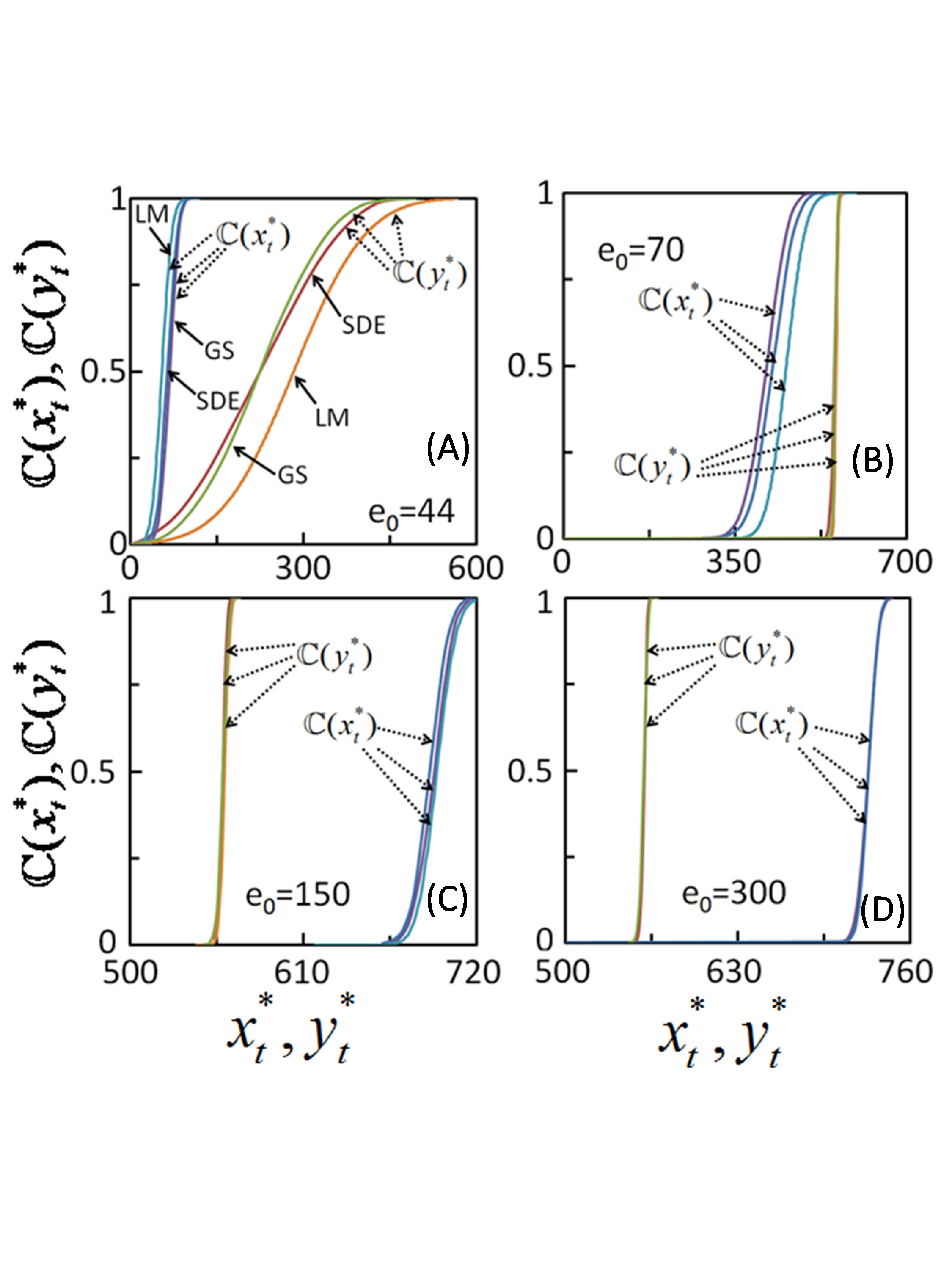

Supplement: Figure S3 — Comparison of the cumulative probability distribution of the stochastic variables and obtained using the three methods viz. , linearization method (LM), stochastic simulations of SDEs (SDE), and Gillespie simulations (GS) for (A) e 0 = 44, (B) e 0 = 70, (C) e 0 = 150 and (D) e 0 = 300. (TIF) [file pone.0035958.s003.tif]
