# Supplementary material for: Noise Propagation in Two-Step Series MAPK Cascade
Source: PLoS One. 2012 May 1;7(5):e35958. doi: 10.1371/journal.pone.0035958 (PMC3341401; doi:10.1371/journal.pone.0035958)
Supplement: Text S1 — Quasi-steady state approximation (QSSA) of the chemical master equation. (DOC) [file pone.0035958.s004.doc]

**Supplementary Text S1. Quasi-steady state approximation (QSSA) of the chemical master equation**

Consider the chemical master equation presented in Eq. (1)

(S1)

where, $\underline{m}=(x_{t},y_{t},xe,x^{*}p_{1},yx^{*},x^{*}p_{2})^{t}$. In order incorporate the QSSA, we assume that the changes in the number of the intermediates occur at a faster time scale than that for the substrates. Therefore, in this faster time scale, we assume [1] that the number of the substrates do not change, that is, $x_{t},y_{t}$ are kept constant in this time scale.

After fixing $\underline{x}=(x_{t},y_{t})^{t}$ in Eq. (S1) and defining $\underline{m}'=(xe,x^{*}p_{1},yx^{*},x^{*}p_{2})^{t}$, we use Bayes theorem for conditional probability to rewrite Eq. (S1) as

(S2)

We then introduce the QSSA by setting the left hand side of Eq. (S2) to zero, that is,

(S3)

Subtracting Eq. (S3) from Eq. (S1) and taking marginal density on the number of the intermediates, we get

(S4)

The expectations in Eq. (S4), obtained using moment generating functions, are (S5)

**Supplementary References**

1. Rao CV, Arkin AP (2003) Stochastic chemical kinetics and the quasi-steady-state assumption: Application to the Gillespie algorithm. J of Chem Phys. 118: 4999-5010.
