# Supplementary material for: Noise Propagation in Two-Step Series MAPK Cascade
Source: PLoS One. 2012 May 1;7(5):e35958. doi: 10.1371/journal.pone.0035958 (PMC3341401; doi:10.1371/journal.pone.0035958)
Supplement: Text S2 — Fourier transform method to estimate noise from linearized SDEs. (DOC) [file pone.0035958.s005.doc]

**Supplementary Text S2. Fourier transform method to estimate noise from linearized SDEs**

The linearized form of the SDEs (Eqs. 5 and 6) is

$\frac{d\Delta x_{t}^{*}}{dt}=-\tau_{1}^{-1}(\Delta x_{t}^{*}-g_{1}\Delta e_{0})+\eta_{1}(t)$ (S1)

$\frac{d\Delta y_{t}^{*}}{dt}=-\tau_{2}^{-1}(\Delta y_{t}^{*}-g_{2}\Delta x_{t}^{*})+\eta_{2}(t)$ (S2)

where, $\tau_{1}^{-1}=\frac{k_{3}K_{1}e_{0}}{(K_{1}+x_{0}-\bar{x}_{t}^{*})^{2}}+\frac{k_{6}K_{2}P_{10}}{(K_{2}+\bar{x}_{t}^{*})^{2}}$ and $\tau_{2}^{-1}=\frac{a_{3}K_{3}\bar{x}_{t}^{*}}{(K_{3}+y_{0}-\bar{y}_{t}^{*})^{2}}+\frac{a_{6}K_{4}P_{20}}{(K_{4}+\bar{y}_{t}^{*})^{2}}$ are the relaxation times, that is the time taken by the system to return the steady state following a perturbation $\Delta x_{t}^{*}$ and $\Delta y_{t}^{*}$. $\bar{x}_{t}^{*},\bar{y}_{t}^{*}$ are the steady number of the phosphorylated substrate for a certain set of parameters. Associated gain factors $g_{1}=\tau_{1}k_{3}(x_{0}-\bar{x}_{t}^{*}/(K_{1}+x_{0}-\bar{x}_{t}^{*})$ and $g_{2}=\tau_{2}a_{3}(y_{0}-\bar{y}_{t}^{*}/(K_{3}+y_{0}-\bar{y}_{t}^{*})$ provide an estimate of the response of the phosphorylated substrate to the fluctuations in the total number of enzyme molecules [1,2].

By taking Fourier transforms of Eqs (S1) and (S2) and applying the inverse Fourier transform, extrinsic and intrinsic noise in both substrates are estimated. Extrinsic noise in the *X*^*^is given by

(S3)

Similarly, extrinsic noise in *Y*^*^is given by

(S4)

Next, the intrinsic noise in *X*^*^ is given by

(S5)

Similarly, the intrinsic noise in *Y*^*^ is given by

(S6)

The first and second terms in Eq. (S6) correspond the intrinsic noise contributions from the first cascade and second cascade, respectively.

**Supplementary References**

1. Detwiler PB, Ramanathan S, Sengupta A, Shraiman BI (2000) Engineering aspects of enzymatic signal transduction: Photoreceptors in the retina. Biophys J 79: 2801-2817.
2. Viswanathan G, Jayaprakash C, Sealfon SC, Hayot, F (2008) Shared kinase fluctuations between two enzymatic reactions. Phys Biol 5: 046002.
