# Supplementary material for: Noise Propagation in Two-Step Series MAPK Cascade
Source: PLoS One. 2012 May 1;7(5):e35958. doi: 10.1371/journal.pone.0035958 (PMC3341401; doi:10.1371/journal.pone.0035958)
Supplement: Text S3 — Comparison of the probability distributions for all three methods. (DOC) [file pone.0035958.s006.doc]

**Supplementary Text S3: Comparison of the probability distributions for all three methods**

In order to compare (for various set of parameters) the extent of the prediction of probability distributions of the linearization method, stochastic simulations of the SDE and Gillespie simulations, we

a) obtained the analytical joint probability distribution of the stochastic variables corresponding to the linearized Langevin equations [1] (Eqs 7 and 8). The joint probability distribution of the stochastic variables corresponding to the linearized Langevin equations (Eqs 7 and 8) is

(S1)

where are respectively the variances of the stochastic variables $x_{t}^{*}$and $y_{t}^{*}$given by Eqs (11) and (12). We then generated the marginal probability distributions for each of the stochastic variables.

b) conducted stochastic simulations of the Langevin model (Eqs 5 and 6) using Euler-Maruyama method [2] for sufficiently large times and obtained 5000 trajectories. We then constructed a histogram of the number of the two stochastic variables.

c) conducted Gillespie simulations [5] and obtained 5000 trajectories. We then constructed a histogram of the number of the two stochastic variables.

Figure (S3) shows a comparison of the cumulative probability distribution obtained for the two stochastic variables using the linearization method (LM), stochastic simulations of the SDE, and Gillespie simulations (GS) for various values of the total upstream enzyme concentration. Note that the distributions are compared in the planes of cumulative probability distribution and the number of substrates in order to circumvent the dependence of the numerically estimated probability distributions on the bin size.

In Figs (S3(A) and (B)), the distributions for the two variables are shown for the parameters in the range (see Fig. 2) where the cascade is very sensitive to the input signal, that is, total upstream enzyme concentration. In this range, while the distribution obtained using the stochastic simulations of the SDE match quite well with Gillespie simulations [3], the linearization method completely fails to predict the distribution of the stochastic variables. In Figs (S3(C) and (D)), the distributions are shown for the parameter range (see Fig 2) where the cascade is not sensitive to the total upstream enzyme concentration. Clearly, the distributions obtained using the three methods agree with each other.

**Supplementary References**

1. Jimenez-Aquino JI (1997) Multivariate formulation of transient stochastic dynamics. Physica A 237: 113-122.
2. Higham DJ (2001) An algorithmic introduction to numerical simulation of stochastic differential equations. SIAM Review 43: 525-546.
3. Gillespie DT (1976) A general method for numerically simulating the stochastic time evolution of coupled chemical reactions. J of Comp Phys 22: 403-434.
